# The effect of wrongdoer’s status on observer punishment recommendations: the mediating role of envy and the moderating role of belief in a just world

**DOI:** 10.3389/fpsyg.2024.1227961

**Published:** 2024-02-15

**Authors:** Zechuan Lin, Fengxiao Cui, Yue Wu, Qingwang Wei

**Affiliations:** ^1^Department of Psychology, Renmin University of China, Beijing, China; ^2^Centre for Mental Health Education, Beijing Vocational Transportation College, Beijing, China

**Keywords:** punishment, social status, envy, belief in a just world, active harm

## Abstract

Our proposition postulates that the correlation between the wrongdoer’s status and the punishment suggestions of onlookers is primarily influenced by group-oriented envy rather than the ascription of intentionality and is moderated by the belief in a just world. In three separate studies, 389 university students were asked to read scenarios describing a hit-and-run crime committed by either a rich or a poor individual and then report their opinions on intentionality attribution (Study 1 and Study 2), envy emotions (Study 2), punishment recommendations (all three studies), and belief in a just world (Study 3). Consistently, the findings indicated that those observing recommended harsher penalties to be imposed upon high-status perpetrators engaging in the same wrongdoing (such as hit-and-run) as their low-status equivalents. The effect of the rich receiving more severe punishment was predicted more strongly by envious emotions than by intentionality attributions to high-status wrongdoers and was only present for those observers who endorsed a lower belief in a just world.

## 1 Introduction

In contemporary times, the chasm between the affluent and the underprivileged has widened, escalating the schism between disparate social classes. This insurmountable class divide is conspicuous in every society worldwide. This study focuses on how attitudes of observers change when there are differences in the social status of the observed individuals and the psychological mechanisms behind it. Specifically, how do observers choose to punish individuals who violate rules when there are differences in social status, and what are the underlying psychological processes? By exploring onlooker’s attitudes and punishment recommendations to the rich wrongdoers, we can understand many social issues related with the consequence of social stratification, such as social perceptions and interactions between different groups, retributive justice, social mentality, and collective action.

Previous researches has shown that we punish the rich more severely. According to the retributive justice literature, intentionality is a key factor in determining the severity of punishment for perpetrators (Jordan et al., 2018; Daumeyer et al., 2019). Novel research has unearthed that those observing the actions of wrongdoers tend to attribute a higher degree of intentionality to the actions of high-status perpetrators, in comparison to the identical actions of those of lower status. This attribution bias often leads to recommendations for more stringent penalties to be imposed upon the former group (Kakkar et al., 2020). However, the stereotype literature also establishes that people often experience upward envy and display hostile prejudice toward high-status group members (Lange et al., 2018). For example, observers may respond with Schadenfreude to the misfortunes of high-status targets (Dasborough and Harvey, 2017). Our argument is that both intentionality attributions and group-based envy may independently influence the severity of punishments recommended for high-status wrongdoers. Furthermore, envy-related hostility is thought to stem from relative deprivation and a sense of injustice in social comparison (Van de Ven et al., 2018). For those who believe in a just world, they may view people as generally receiving what they deserve, and thus treat different wrongdoers equally (Bartholomaeus and Strelan, 2019). Therefore, we also propose that the tendency to punish high-status wrongdoers more severely is for those observers who endorsed a lower belief in a just world.

## 2 Conceptual framework

### 2.1 Observer punishment and wrongdoer status

The phenomenon of more severe punishment for high-status wrongdoers cannot be confirmed without comparing the punishment recommended by participants for high-status wrongdoers to that recommended for low-status wrongdoers who have committed an identical crime. Retributive justice research provides a paradigm of observer punishment, which focuses on the blame and punishment judgments of lay observers for an identical misbehavior by varying the level of perpetrator intention and the harm caused by the misbehavior (Jordan et al., 2018; Daumeyer et al., 2019). Previous research has demonstrated that this paradigm is useful for exposing intergroup bias by varying the identities of perpetrators (Kung et al., 2016). Those who hold negative attitudes toward the rich may conceal their hostile feelings and intentions unless the rich make a mistake. Therefore, the punishment judgments can allow biases against the rich to be expressed under the guise of justice, as punishing a wrongdoer is considered socially desirable behavior. Thus, we have chosen the paradigm of observer punishment to explore the rancor against the rich by varying the wrongdoers’ status (high or low).

The severity of punishment for criminal behavior is often influenced by the perceived level of intentionality, with intentional behavior resulting in more severe punishment (Jordan et al., 2018; Daumeyer et al., 2019; Picó et al., 2020; Yao and Siegel, 2021). Research has demonstrated that observers typically assign a greater degree of intentionality to the actions of high-status individuals, compared to the actions of those of lower status, even when the wrongdoing is identical. This attribution bias frequently results in marked discrepancies in punishment recommendations (Kakkar et al., 2020). However, research on the evaluation of workplace misbehavior has found the opposite effect, with lower-status actors being evaluated more harshly than higher-status actors (Polman et al., 2013; Blue et al., 2018). The “protection effect” afforded to high-status individuals is more conspicuous in cases of minor misbehavior, but it is not apparent in incidents of severe wrongdoing (Karelaia and Keck, 2013). It is speculated that the societal connection between the affluent and the underprivileged is less transactional than the relationship between individuals of high and low status within organizations. Moreover, in retributive justice research, punishment judgments are often driven not only by moral outrage, but also by dehumanization (Bastian et al., 2013). The Stereotype Content Model (SCM), introduced by Cuddy et al. (2018a,b,c), posits that competence and warmth are the two core dimensions of social perception, and that social stereotypes and emotions frequently stem from these dimensions. In this model, high-status individuals or groups are often stereotyped as being competent but lacking in warmth which suggests a tendency to punish the rich more severely, with a focus on the role of intentionality attribution in this tendency. Therefore, we hypothesized that intentionality attribution mediates the relationship between wrongdoer’s status and onlookers’ punishment.

### 2.2 Envious prejudice against high status groups

In SCM, high-status individuals or groups’ competent-cold stereotype can trigger the emotion of envy. Envy is an ambivalent emotion characterized by feelings of resentment, hostility, and inferiority that arises from negative social comparisons with other individuals or groups. The wealthy are often viewed as an envied group (Wu et al., 2018; Cuddy et al., 2018a,b,c). Recent studies have shown that many Chinese people identify themselves as lower status than their objective status, which may lead to greater relative deprivation and, consequently, more envy towards the wealthy in Chinese society (Yu et al., 2019).

Thus, envy may be an important factor in understanding why the rich are often punished more severely than others. The SCM provides a useful framework for exploring the ambivalent stereotypes and emotions that people hold towards high-status individuals, which may ultimately shape our attitudes towards them and their behavior. In addition, understanding these dynamics in different cultural settings is important for developing a more nuanced understanding of how perceptions of status and wealth shape social interactions and attitudes.

Previous studies have provided ample evidence for the relationship between envious emotions and hostile behaviors (or intentions) (Hofer and Busch, 2011; Kim and Glomb, 2014; Van de Ven et al., 2018). For example, individuals who experience envy are often motivated to actively denigrate those who they perceive as superior (Rentzsch et al., 2015). Furthermore, envy can lead to sabotaging and attacking higher status “groups” (Cuddy et al., 2018a,b,c), with Schadenfreude, or joy in another’s misfortune, often directed at high status groups in particular (Dasborough and Harvey, 2017).

Moreover, recent evidence has suggested that envious emotions more strongly and directly predict behaviors than the competent-cold stereotype, which has been considered as the basis of intentionality attribution (Cuddy et al., 2018a,b,c). These findings highlight the importance of considering envy as a potential underlying factor in hostile behaviors and attitudes towards high-status individuals or groups, and suggest that interventions targeting envy may be effective in reducing such behaviors.

The rich constitute a salient superior group, and envy is a form of harm waiting to happen in contemporary society. This assertion is supported by numerous examples of social unrest and conflict that can be traced back to the unequal distribution of wealth and resources. The most of intergroup conflict incidents occurred in China are derived from interpersonal conflicts between the low status and high status (Wang, 2009). The case of Yao Jiaxin occurred in 2010–2011 showed the power of public opinion based on this group emotion. Yao was a 21-year-old student who accidentally struck a young poor peasant woman with his car and silenced her by stabbing her to death on the roadway. Although Yao surrendered to the police, which would usually result in no-death sentence, he was eventually executed under the pressure of public resentment against his identity of the “fu er dai,” the “rich second generation” of privileged families (Wines, 2011) Like Yao case, Chinese public opinions claim to punish a rich person who signal envied group more severely once he make some misbehaviors. Most incidents of intergroup conflict arise from interpersonal conflicts between individuals of low and high status. It is argued that the more severe punishments recommended for high-status wrongdoers are manifestations of discriminatory behavior. It is suggested that observers’ recommendations for more stringent penalties for high-status wrongdoers are influenced more strongly by their envious emotions towards the wrongdoers as a group than by their attributions of intentionality to the wrongdoers as individuals.

Thus we hypothesized that both intentionality attributions and group-based envy independently influence punishment severity, with group-based envy exerting a stronger effect on punishment recommendation.

### 2.3 The belief in a just world

Both the attribution of intentionality and the envy response to high-status wrongdoers reflect a shared motive for punishment, namely the justice motive. A higher attribution of intentionality signifies a greater degree of responsibility and moral wrongdoing on the part of the targets for their actions, resulting in a correspondingly more significant punishment proportionate to the severity of their actions. Similarly, an envious response to high-status wrongdoers can lead to a desire for justice, as observers may seek to address perceived inequalities and level the playing field between high and low-status groups (Crockett et al., 2014). The hostility associated with envy is argued to arise from feelings of relative deprivation and a sense of injustice in social comparisons (van de Ven and Zeelenberg, 2020). In situations of resentment against the rich, we propose that the judgment of wrongness in attribution of intentionality is based more on the individual’s actions, while the unjust feeling involved in the envy response is based more on the group identity of the wrongdoer. However, individuals may differ in both their emotional tendency towards envy and their beliefs regarding retributive justice (Crusius and Lange, 2017; Osgood, 2017; Van de Ven et al., 2018).

The belief in a just world (BJW) is a common belief that individuals live in a world that is inherently just and that people generally receive what they deserve and deserve what they receive (Lerner, 1980). BJW serves as a psychological need to believe in a just world and shapes how people respond to issues of justice and injustice. It often motivates individuals to blame victims for their fate and cast aspersions on their character (Bartholomaeus and Strelan, 2019). However, it also compels individuals to uphold justice rationally to maintain their belief in a just world when dealing with affairs (Bartholomaeus and Strelan, 2019). Recent studies have indicated that BJW is linked to a strong inclination towards making long-term investments and a strong aspiration to achieve socially desirable goals through socially acceptable means (Hafer et al., 2005; Hafer and Sutton, 2016). Individuals who believe in a just world tend to be more optimistic about the future and have faith that hard work and dedication will ultimately lead to success. Furthermore, they are more likely to engage in behaviors that are socially responsible and ethical, as they believe that doing so will ultimately lead to a just outcome. These findings suggest that BJW plays a significant role in shaping people’s values and behaviors, particularly in their pursuit of long-term goals. Most recent research has focused on explicitly endorsed individual differences in BJW (Osgood, 2017). For individuals who strongly endorse BJW, it can serve as a psychological resource to buffer negative feelings triggered by unjust events (Bartholomaeus and Strelan, 2019). Conversely, those who weakly endorse BJW have a hostile attributional bias (Bartholomaeus and Strelan, 2019). We infer that high BJW can lead to equal punishment recommendations for different transgressors who commit identical crimes. Therefore, individuals who have a higher belief in a just world may punish high-status and low-status wrongdoers equally but more severely. So we hypothesized that the link between wrongdoers’ status and the extent of punishment proposals would be contingent on the observers’ belief in a just world.

### 2.4 Current research

The current studies are the first to systematically investigate the phenomenon of rancor directed towards affluent individuals through controlled experimental designs in China, making them particularly valuable to the field. Participants in three studies engaged in observer punishment, wherein they were presented with scenarios describing a hit-and-run crime committed by either a wealthy or impoverished individual, which bears relevance to recent incidents discussed within Chinese society. We differentiated between high status that is ascribed versus achieved (Study 2) and measured participants’ attributions of intentionality (Studies 1 and 2), experience of envy (Study 2), and belief in a just world (Study 3). Our hypothesized models propose that both intentionality attributions and group-based envy independently influence punishment severity, with group-based envy exerting a stronger effect on punishment recommendation. Moreover, we anticipate that the link between wrongdoers’ status and the extent of punishment proposals would be contingent on the observers’ belief in a just world. The present findings offer valuable insights into the complex interplay of social factors influencing punishment outcomes in China.

## 3 Study 1: wrongdoer’s social status and punishment recommendations

### 3.1 Methods

For Study 1, participants were presented with a hypothetical scenario involving illegal behavior, such as a hit-and-run incident. The purpose was to investigate the effect of the wrongdoer’s social status (high vs. low) on observers’ attribution of responsibility and their recommended severity of punishment. Drawing on the findings of Kakkar et al. (2020) with Western participants, we predicted that participants would advocate harsher punishment for the high-status wrongdoer. Additionally, we posited that the connection between the perpetrator’s societal status and the suggested punishments would be mediated by the degree of intentionality ascribed to the offender.

#### 3.1.1 Participants

A convenience-based cluster sampling of 148 college students was recruited from Beijing (60.1% females). Informed consent was obtained from all participants in the study. The participants were predominantly aged between 18 and 32 years (,*M_age_* = 21.84 years, *SD* = 2.41). We collected information about their subjective social status (MacArthur Scale), enabling us to control for potential confounding variables related to social status and its influence on punishment recommendations. The questionare is paper-based and was gathered in classroom or laboratory.

#### 3.1.2 Procedure

Each individual participant was invited to complete an anonymous survey that pertained to their social attitudes. Prior to commencing the survey, they were acquainted with the study protocols and gave their consent. Participants were subsequently assigned to the high or low target social status group through randomization. Afterward, they reviewed a hypothetical scenario and completed a manipulation check to assess their perception of the wrongdoer’s social status. Additionally, they provided intentionality attributions, punishment recommendations, and demographic information. Upon the survey’s completion, participants were debriefed and given tokens as a gesture of appreciation for taking part in the study.

#### 3.1.3 Materials, measures and procedures

The participants in this current investigation were directed to review a theoretical scenario that depicted unlawful conduct, such as a hit-and-run incident. Specifically, *X hit a person while driving through an intersection one night, but did not stop to identify himself. Two days later, the police located X and notified them that the victim was still hospitalized and there was a possibility of permanent disability. X was subsequently accused of hit-and-run.* In order to manipulate social status, we varied the target’s family background (i.e., second-generation rich or second-generation migrant worker) and occupation (i.e., high-status CEO of a family business with his own car, or low-status worker in a small construction company who drove part-time for the company). These manipulations were based on research about class structure in contemporary Chinese society (Lu, 2002).

To ensure that our manipulations were successful, participants were asked to rate the target’s education level, occupation status, personal income, and societal status on a 7-point scale (1 = *very low*; 7 = *very high*). The four items were combined to generate a composite measure of social status by taking the average score (*α* = 0.89).

*Intentionality attributions* were assessed using two items: “The hit-and-run driver did this on purpose” and “The hit-and-run driver did this because of negligence.” Participants rated the likelihood of each attribution on a 7-point scale (1 = *absolutely impossible*, 7 = *absolutely possible*). The second item was reverse-coded so that higher scores indicated greater attribution of intentionality. The two items were averaged to create a composite measure of intentionality attributions (α = 0.63).

*Punishment Recommendations* In order to assess punishment recommendations, we focused on retributive punishment as a better indicator of “hate rich” attitudes [corresponding to active harm in Wu et al. (2018)]. The participants were requested to indicate their level of agreement with two items (e.g., “*X should be sued for hit-and-run*” and “*He should be subject to criminal liabilities according to law*”) on a 7-point scale (1 = *strongly disagree*, 7 = *strongly agree*).

Overall, these measures were used to examine the effect of the target’s social status on observers’ attribution of responsibility and recommended severity of punishment for the hit-and-run incident.

### 3.2 Results

#### 3.2.1 Manipulation check

Target in the “rich second generation business owner” condition was judged to have higher status than that in the “second generation migrant worker” condition (*M_high_* = 4.63, *SD_high_* = 0.83; *M_low_* = 2.58, *SD_low_* = 0.63; *t* (146) = 16.76, *p* < 0.001). This finding indicated that the manipulation of social status worked as intended.

#### 3.2.2 Effects of social status on intentionality attributions and punishment recommendations

**Figure 1:**
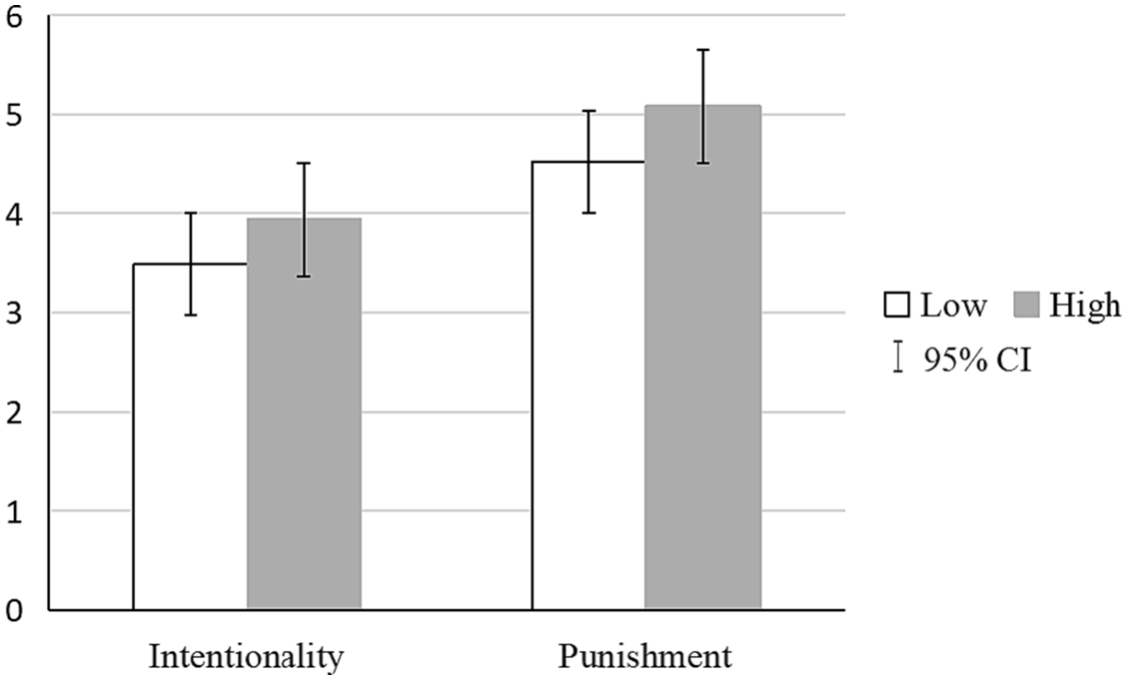
Status differences in intentionality attributions, and punishment severity.

Table 1 and Figure 1 depict the descriptive statistics and correlation among the wrongdoer’s status, intentionality attributions, and punishment recommendations. The observer’s perception of the wrongdoer and their recommended punishment were influenced by the target status group they were assigned to. Consistent with our hypothesis, participants rated significantly higher on intentionality attributions when the hit-and-run driver was described as a high (*M_high_* = 3.93, *SD_high_* = 0.94), as opposed to low (*M_low_* = 3.49, *SD_low_* = 0.98) status individual (*t* (146) = 2.80, *p* = 0.006, *d* = 0.46). Moreover, the participants suggested a more severe punishment when the target was portrayed as a high-status (*M_high_* = 5.08, *SD_high_* = 0.96) individual as compared to when they were depicted as a low-status (*M_low_* = 4.52, *SD_low_* = 1.01) individual (*t* (146) = 3.47, *p* = 0.001, *d* = 0.57).

**Table 1:** Descriptive statistics of key variables in Study 1.

	*M*	*SD*	Wrongdoer’s status	Intentionality attributions
Wrongdoer’s status	–	–		
Intentionality attributions	3.73	0.98	0.23**	
Punishment severity	4.82	1.02	0.28**	0.21^**^

^**^p < 0.01.

#### 3.2.3 The mediation role of intentionality attributions

**Figure 2:**
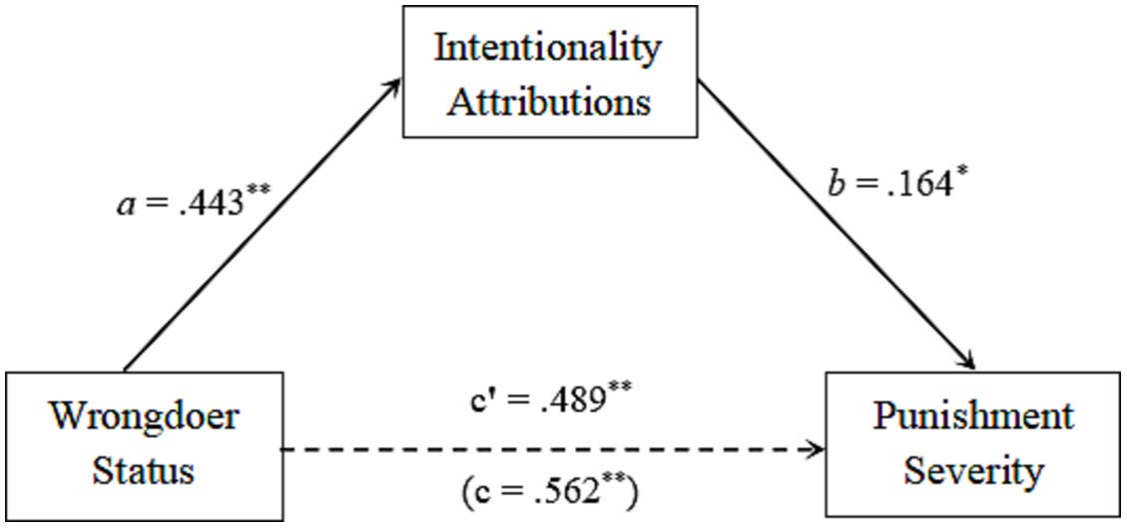
Mediated effect of intentionality attributions; ^*^ *p* < 0.05, ^**^ *p* < 0.01.

Structural equation modeling, specifically path analysis, was utilized to examine the hypothesis that intentionality attributions acted as a mediator between the wrongdoer’s social status and the recommended punishment. Mediation is considered to exist if the magnitude of the indirect effect is significantly different from zero (Shrout and Bolger, 2002). In this case, The indirect effect is the impact of the wrongdoer’s social status on the recommended punishment through the mediator of intentionality attributions (path a * path b in Figure 2).

To estimate the magnitude of the indirect effect, we employed the AMOS 28.0 bootstrap technique. This procedure entailed randomly generating 5,000 subsamples with replacement from the complete dataset (Stine, 1989; Efron and Tibshirani, 1993). The indirect impact in the original dataset was calculated as 0.443*0.164 = 0.073. The bootstrap procedure produced a 95% confidence interval for the indirect effect that did not include zero, ranging from 0.003 to 0.202. This finding implies that the indirect effect was significantly different from zero.

In addition, the direct effect of the wrongdoer’s social status on the recommended punishment was also statistically significant (direct effect was 0.003). As a result, it appears that intentionality attributions played a partial role in mediating the association between the wrongdoer’s social status and the suggested punishment. High-status wrongdoers were perceived to have more intentionality for the same transgression as opposed to low-status wrongdoers. Furthermore, we found that as participants perceived the target’s transgression to be more intentional, their agreement with recommending more severe punishment also increased. The indirect impact accounted for 13% of the total effect, suggesting that the intentionality mediating process explained 13% of the effect of social status on punishment.

### 3.3 Discussion

Consistent with prior research (Dong et al., 2022), The findings of Study 1 revealed that participants suggested more severe punishments and associated greater intentionality with the same wrongdoing when the perpetrator was identified as high-status, in comparison to low-status. Additionally, the outcomes showed that intentionality attributions played a partial mediating role in the relationship between social status and punishment severity, but only explained 13% of the total effect. Controlling for this mediator, the status effect on punishment recommendations was still significant. Therefore, other mechanisms (mediators) may explain why high-status wrongdoers tend to be punished more severely.

According to the Social Cognitive Model and BIAS theory, emotions may play a stronger role in shaping behavior than cognitive variables (Wu et al., 2018). Previous research has suggested that envy, as a typical affective response towards high-status individuals (characterized as high competence but low warmth), may elicit harmful behavior (Wu et al., 2018). Thus, in Study 2, we assessed participants’ feelings of envy towards individuals like the target, in addition to intentionality attributions. In our study, we examined the potential mediating effects of both envy and intentionality attributions on punishment recommendations, and explored whether envy (as an affective mediator) can better explain the effect of social status on punishment severity than attributions of intentionality (as a cognitive mediator).

Another interesting question that remains to be explored is whether the evidence of hatred towards the rich found in Study 1 can be extended to high-status individuals who achieve their status through personal efforts, such as education and hard work. Some second-generation wealthy individuals are known for their extravagant lifestyle and have become targets of social hatred towards the rich. However, do people hold similar attitudes towards individuals who attain high status through their own efforts? To address this issue, Study 2 distinguished between second-generation and self-made rich individuals and compared the punishment recommendations for these two groups.

## 4 Study 2: mediation role of envy emotions

### 4.1 Methods

Study 2 extended study 1 in two ways. Firstly, we included envy emotions in our analysis to investigate the mechanisms that underlie the association between social status and punishment recommendations. We hypothesized that individuals would experience greater envy towards high-status wrongdoers, which would predict more severe punishment recommendations. Given the prominent role of emotions in shaping behavior (Jones et al., 2017; Wu et al., 2018; Radke et al., 2020), we anticipated that envy emotions would offer a more compelling explanation for the phenomenon of hatred towards the rich than intentionality attributions (as a cognitive mediator). Secondly, we conducted an exploratory analysis to distinguish between second-generation (e.g., inherited wealth) and self-made (e.g., education, occupation) high-status individuals.

#### 4.1.1 Participants

A convenience-based cluster sampling of 116 college students was recruited from Beijing (57.8% females). Informed consent was obtained from all participants in the study. The participants were predominantly aged between 18 and 43 years (*M_age_* = 21.16 years, *SD* = 3.15). We collected information about their subjective social status (MacArthur Scale), enabling us to control for potential confounding variables related to social status and its influence on punishment recommendations. The questionare is paper-based and was gathered in classroom or laboratory.

#### 4.1.2 Materials, measures and procedures

We manipulated the target status by providing different information regarding their family background, education, and occupation. In the present study, we operationalized high-status in two distinct ways. In the second-generation high status condition (ascribed), we described the target as a wealthy individual who inherited their wealth and held the CEO position in their family business. In the self-made high status condition (achieved), we described the target as a highly educated individual who had earned a master’s degree from a prestigious university and was currently working as a department manager in a large corporation. In contrast, in the low-status condition, the target was described as a second-generation migrant worker who worked as a driver for a small company.

*Envy emotions* to measure envy emotions, we asked participants to rate, on a 6-point scale, the extent to which they experienced feelings of envy and jealousy towards individuals similar to the target described in each scenario. Envy and jealousy are typical emotions people feel towards the envied group (e.g., the rich), according to SCM and BIAS theory (Wu et al., 2018). After participants responded to the envy and jealousy items, we summed their scores on both items and calculated an average score. Higher scores on this measure indicated that participants experienced more intense envy emotions towards individuals similar to the target described in each scenario (α = 0.73).

*Punishment recommendations* was assessed using the same item (α = 0.85) from Study 1.

*Perceived target status and intention attributions* were using the same items as in Study 1 (αs = 0.83 and 0.73 for perceived status and intentionality, respectively).

The protocols in Study 2 were analogous to those in Study 1. Participants were assigned randomly to one of three conditions that varied by target status (second-generation high, self-made high, and low status). After reading the same hypothetical scenario used in Study 1, the participants filled out an array of measures to evaluate their perceptions of the target’s status, their experience of envy emotions towards individuals similar to the target, their attributions of intentionality, their severity recommendations for punishment, and their demographic information.

### 4.2 Results

#### 4.2.1 Manipulation check

Participants gave relatively high ratings on perceived social status to target in the second-generation high (*M* = 4.37, *SD* = 0.57) status and self-made high (*M* = 4.63, *SD* = 0.58) status conditions, compared to that in the low (*M* = 2.70, *SD* = 0.55) status condition (*F* (2, 113) = 127.76, *p* < 0.001, *η^2^* = 0.69). Bonferroni *post hoc* tests revealed that the differences in perceived status were statistically significant between each of the high-status conditions and the low-status condition (*p* < 0.001), whereas the perceived status score did not differ between the second-generation high and self-made high status groups. These findings indicated that the manipulation of social status worked as intended.

#### 4.2.2 Envy emotions, intentionality attributions, and punishment recommendations for wrongdoers of different status

Table 2 showcases the descriptive statistics for the wrongdoer’s status, envy emotions, attributions of intentionality, and punishment recommendations. The results indicated that wrongdoer’s status, envy emotions, and punishment recommendations were positively correlated with each other. Furthermore, intentionality attributions were positively correlated with punishment recommendations, but not significantly correlated with the other two variables. Importantly, the correlation between envy and punishment recommendations was found to be stronger than the correlation between attributions of intentionality and punishment recommendations.

**Table 2:** Descriptive statistics of key variables in Study 2.

	*M*	*SD*	Wrongdoer’s status	Envy emotions	Intentionality attributions
Wrongdoer’s status	–	–			
Envy emotions	2.79	1.11	0.62^**^		
Intentionality attributions	3.71	0.96	0.16^**^	0.12^**^	
Punishment severity	4.70	1.03	0.50**	0.50**	0.22^**^

^**^p < 0.01.

Next, we tested status differences in envy emotions, intentionality attributions, and punishment recommendations using one-way ANOVA. Participants reported significantly more envy emotions toward the high (*M_ascribed_* = 3.36, *SD_ascribed_* = 0.81; *M_achieved_* = 3.24, *SD_achieved_* = 0.90) status group compared to that toward the low (*M_low_* = 1.65, *SD_low_* = 0.67) status group (*F* (2, 113) = 52.46, *p* < 0.001, *η*^2^ = 0.48). *Post hoc* test (LSD) showed that the difference in envy emotions was significant between each of the high status condition and the low status condition (*M_ascribed_* = 3.36 > *M_low_* = 1.65, *p* < 0.001; *M_achieved_* = 3.24 > *M_low_* = 1.65, *p* < 0.001). People’s envy emotions toward the two high status groups (i.e., ascribed and achieved) did not differ significantly (*M_ascribed_* = 3.36 > *M_achieved_* = 3.24, *p* = 0.49).

However, intentionality attributions toward different status groups (i.e., high and low) did not differ significantly (*M_ascribed_* = 3.90, *SD_ascribed_* = 1.01; *M_achieved_* = 3.69, *SD_achieved_* = 0.98; *M_low_* = 3.53, *SD_low_* = 0.86), missing variance with Welch test method (Welch *F* (2, 113) = 2.15, *p* = 0.15). *Post hoc* test (Games-Howell) showed that the status differences in intentionality attributions were not significant between each of the high status condition and the low status condition (*M_ascribed_* = 3.90 > *M_achieved_* = 3.69, *p* = 0.32; *M_ascribed_* = 3.90 > *M_low_* = 3.53, *p* = 0.09; *M_achieved_* = 3.69 > *M_low_* = 3.53, *p* = 0.47).

For punishment severity, participants endorsed more severe punishment when the wrongdoer was in the high (*M_ascribed_* = 5.19, *SD_ascribed_* = 0.93; *M_achieved_* = 4.91, *SD_achieved_* = 0.99) status condition than when the target was in the low (*M_low_* = 3.92, *SD_low_* = 0.93) status condition (*F* (2, 113) = 21.32, *p* < 0.001, η^2^ = 0.22). *Post hoc* test (LSD) showed that the difference in punishment recommendations was significant between each of the high status condition and the low status condition (*M_ascribed_* = 5.19 > *M_low_* = 3.92, *p* < 0.001; *M_achieved_* = 4.91 > *M_low_* = 3.92, *p* < 0.001). People’s punishment recommendations toward the two high status groups (i.e., ascribed and achieved) did not differ significantly (*M_ascribed_* = 5.19 > *M_achieved_* = 4.91, *p* = 0.17).

**Figure 3:**
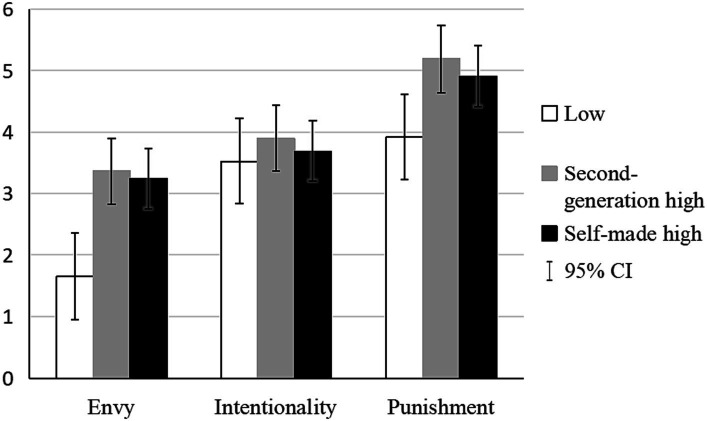
Status differences in envy emotions, intentionality attributions, and punishment severity.

Table 2 and Figure 3 illustrate the mean scores of envy emotions, attributions of intentionality, and punishment recommendations for each status condition.

#### 4.2.3 Testing mediated effects of envy emotions and intentionality attributions

**Figure 4:**
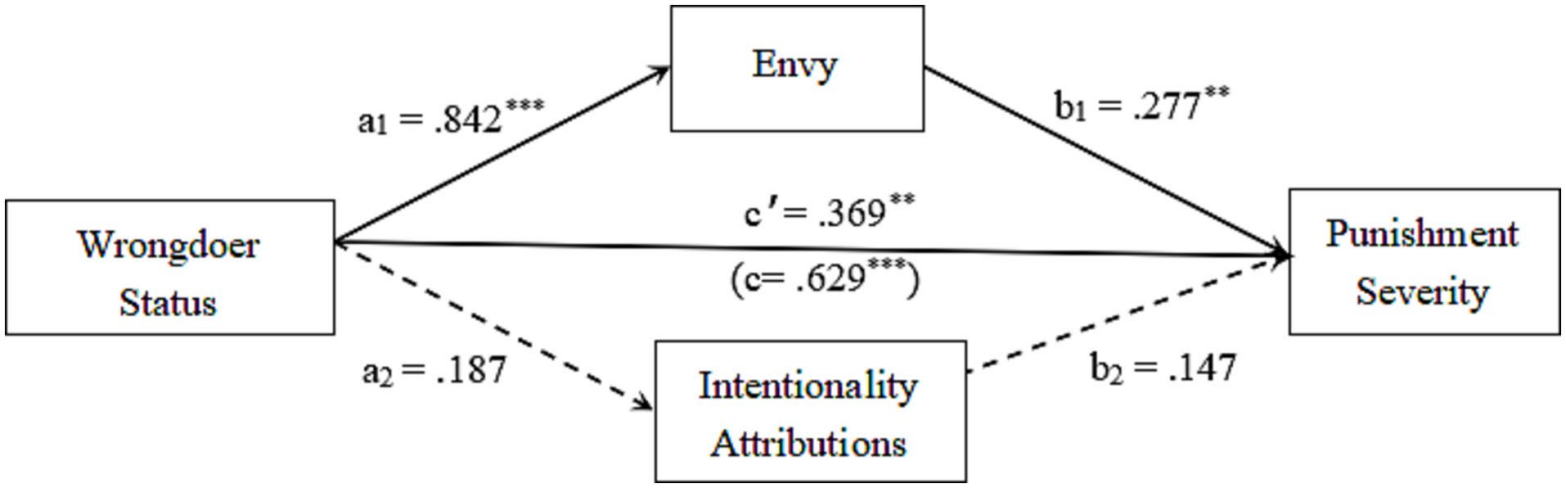
Testing mediated effects of envy emotions & intentionality attributions. ^**^ *p* < 0.01, ^***^ *p* < 0.001.

Following Beardsley’s (2017) recommendations, we utilized path analysis to examine the parallel mediator model presented in Figure 4. Since there were no significant differences in envy emotions, intentionality attributions, and punishment recommendations scores between the second-generation and self-made high status conditions, we combined these groups to create a single high status group for the mediation analysis.

Similarly to Study 1, we performed a bootstrap procedure using 5,000 random samples with replacement from the entire sample to assess the size of indirect effects in the model. As displayed in Figure 4, the overall effect of the wrongdoer’s status on punishment recommendations was 0.629, and the 95% confidence interval (CI) did not encompass zero (0.424, 0.841), indicating a significant total effect. This significant total effect was distributed over three paths, including one direct and two indirect paths, in the proposed model.

The indirect effect through envy emotions was 0.842*0.277 = 0.233, and the 95% C.I. of this indirect effect excluded zero (0.072, 0.396), signifying a significant mediation effect through envy emotions. This indirect pathway explained 37% of the basic relationship between wrongdoer’s status and punishment recommendations.

However, the indirect effect through intentionality attributions was 0.187*0.147 = 0.027, and the 95% C.I. of this indirect effect included zero (−0.003, 0.120), indicating a non-significant indirect effect through attributions of intentionality. This indirect pathway accounted for only 4% of the basic status-punishment linkage.

Based on the findings of the study, we can conclude that only envy emotions partially mediated the connection between status and punishment recommendations when both envy emotions and intentionality attributions were simultaneously taken into account in the path model.

### 4.3 Discussion

Study 2 replicated the finding from Study 1 that high status wrongdoers were punished more severely. This study has contributed to advancing the understanding of the psychology of prejudice by highlighting the mediating role of envy emotions in the relationship between status and punishment recommendations. Specifically, people felt more envy towards the high status group, which resulted in harsher punishment for high status transgressors. Consistent with our hypothesis, envy emotions explained a larger proportion of the basic relationship between status and punishment recommendation than did intentionality attributions. In previous research on intentionality attribution and punishment recommendations, the context and status of the target were often ignored (Jordan et al., 2018; Daumeyer et al., 2019; Picó et al., 2020; Yao and Siegel, 2021). As a result, observers’ recommendations for the severity of punishment were based solely on the behavioral act itself, with intentionality being seen as an integral part of the behavior. This study used a punishment paradigm and introduced contextual variables such as the wrongdoer’s status. When the behavior is consistent, observers’ recommendations for punishment severity are more influenced by the wrongdoer’s identity information. Study 2 introduced the emotion of jealousy, and the different status of the target led to differences in the level of jealousy, which influenced the impact of intentionality attribution on punishment severity.

Moreover, Study 2 revealed an interesting pattern: people tend to recommend more punishment on high status wrongdoers regardless of whether their social status was second-generation or self-made. This begs the question: is “hatred of the rich” a robust and universal phenomenon? Additionally, are there individual difference variables that may influence people’s punitive judgments of high versus low status wrongdoers? Previous research on belief in a just world (BJW) suggests that this may be the case, as BJW functions as a psychological buffer that helps individuals maintain mental health and trust in the fairness of the world (Nudelman and Nadler, 2017).

In Study 3, we examined belief in a just world (BJW) as a moderator of the effect of status on punishment recommendations. Specifically, we hypothesized that individuals with lower levels of belief in a just world would recommend more severe punishment when the wrongdoer was of high status compared to low status. Conversely, individuals with higher levels of belief in a just world would endorse similar punishment for the identical transgression, regardless of the wrongdoer’s status.

## 5 Study 3: moderation effect of BJW

The objective of Study 3 was to replicate prior investigations on “anti-rich sentiment” and explore the moderating function of belief in a just world (BJW) in the association between social status and punishment recommendations. Findings from an experiment utilizing hypothetical scenarios and a BJW assessment revealed that people with diminished BJW advocated for more severe punishment for high-status perpetrators. These findings provide insight into the impact of BJW on social justice and intergroup relations.

### 5.1 Methods

#### 5.1.1 Participants

A convenience-based cluster sampling of 116 college students was recruited from Beijing (59.2% females). Informed consent was obtained from all participants in the study. The participants were predominantly aged between 19 and 36 years (*M*_age_ = 22.50 years, *SD* = 2.84). We collected information about their subjective social status (MacArthur Scale), enabling us to control for potential confounding variables related to social status and its influence on punishment recommendations. The questionare is paper-based and was gathered in classroom or laboratory.

#### 5.1.2 Materials, measures and procedure

In keeping with Study 1, the hypothetical hit-and-run scenario and manipulation of target status were employed.

*Punishment recommendation* was assessed using the same item (*α* = 0.85) from Study 1.

*Perceived target status* was measured using the same four items (*α* = 0.85) from Studies 1 and 2.

*Belief in a Just world* was evaluated using an eight-item BJW for others (BJW-O) subscale from the BJW scale (Lipkusa et al., 1996). A sample item was “I feel that people get what they deserve.” Response options ranged from 1 (*strongly disagree*) to 6 (*strongly agree*), where higher scores reflected stronger BJW (α = 0.73).

The procedure of Study 3 was similar to that of Study 1. Participants first read the hypothetical scenario, then filled out items on perceived status of the wrongdoer, punishment recommendations, BJW and demographic information.

### 5.2 Results

#### 5.2.1 Manipulation check

Target in the “rich second generation business owner” condition was judged to have higher status than that in the “second generation manual worker” condition (*M_high_* = 4.45, *SD_high_* = 0.59; *M_low_* = 2.81, *SD_low_* = 0.45; *t* (123) = 16.81, *p* < 0.001). This finding indicated that the manipulation of social status worked as intended.

#### 5.2.2 Punishment recommendations

Similar to Studies 1 and 2, participants suggested stricter penalties when the subject was portrayed as having high (*M_high_* = 4.90, *SD_high_* = 0.92) social status compared to when the subject was depicted as having low (*M_low_* = 4.17, *SD_low_* = 1.07) social status (*t* (123) = 4.09, *p* < 0.001, *d* = 0.73). As shonw in Table 3, wrongdoer’s status, BJW scores and punishment recommendation significantly correlated with each other pairwise. Specifically, there was significant positive correlation between BJW scores and punishment recommendation (*r* = 0.23, *p* = 0.007). These outcomes suggest that individuals who possess stronger BJW are inclined to support more stringent punishments for wrongdoers.

**Table 3:** Descriptive statistics of key variables in Study 3.

	*M*	*SD*	Wrongdoer’s status	Punishment severity
Wrongdoer’s status	–	–		
Punishment severity	4.59	1.06	0.39^***^	
BJW	3.54	0.56	0.20^*^	0.23^**^

^*^p < 0.05; ^**^p < 0.01; ^***^p < 0.001.

#### 5.2.3 Moderation effect of BJW

**Figure 5:**
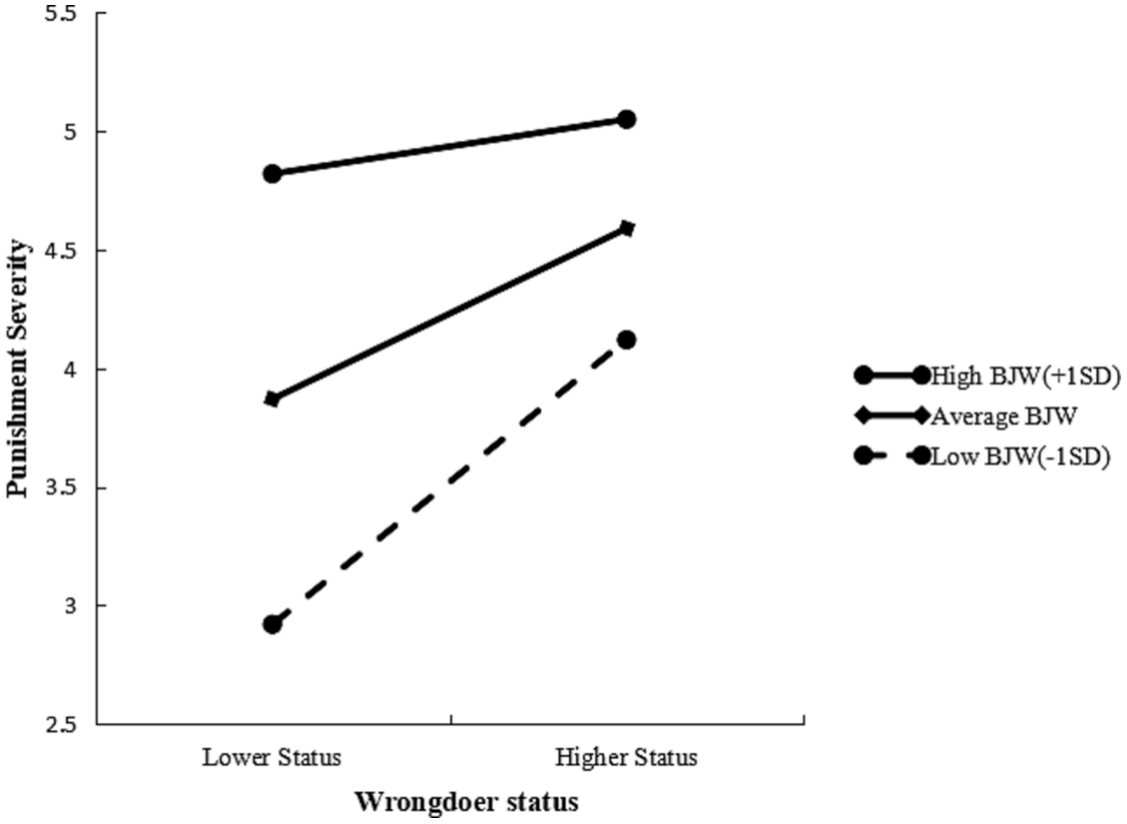
Simple slope test of the moderating effect of BJW.

The present investigation examined the hypothesis of BJW’s moderating impact on the association between perpetrator status and punishment recommendation. The regression technique outlined by Aiken et al. (1991) was utilized to test this hypothesis. At step 1 of the hierarchical regression model, participants’ age and gender (0 = male) were entered as control variables. At step 2, main effects for wrongdoer status (0 = low, 1 = high) and BJW scores were included, and two-way interactions between social status and BJW scores were added at step 3. Table 4 presents the regression results. Multiple linear regression analysis was conducted utilizing SPSS, with punishment recommendation as the dependent variable, wrongdoer status as the independent variable, BJW as the moderator, and grade, age, and gender as control variables. The findings revealed that individuals with higher BJW scores administered comparable punishment to both high-status and low-status wrongdoers (*B* = −0.861, *t* = −2.53, *p* = 0.013, *ΔR*^2^ = 0.046). As displayed in Figure 5, the tendency to punish high status wrongdoers more severity weakened as the observer held stronger BJW. In fact, the status effect on punishment severity was only documented among people who scored low (*B* = 1.17, *p* < 0.001) and moderate (*B* = 0.70, *p* < 0.001) on the BJW scale, whereas for people who scored high on the BJW scale, the wrongdoer’s status did not affect their judgment of punishment severity (*B* = 0.23, n.s.).

**Table 4:** Testing moderated effects of BJW.

Predictor	Punishment severity			
	1st step	2nd step	3rd step	4th step
Age	−0.00^***^	−0.01^***^	−0.01^***^	0.00^***^
Gender	0.04^***^	0.09^***^	0.07^***^	0.01^***^
Grade	0.02^***^	0.01^***^	0.03^***^	0.07^***^
Wrongdoer status		0.84^***^	0.77^***^	3.76^***^
BJW			0.32^***^	1.69^***^
Wrongdoer status × BJW				−0.86^***^
Adjusted *R*^2^	−0.03^***^	0.12^***^	0.14^***^	0.18^***^
*ΔR*^2^	0.00^***^	0.15^***^	0.03^***^	0.05^***^

^*^p <0.05; ^**^p < 0.01; ^***^p < 0.001.

### 5.3 Discussion

The present study’s results replicated those of Study 1 and Study 2, demonstrating that participants recommended more severe punishment for high status targets than for low status targets. Additional analyzes examining Belief in a Just World (BJW) unveiled a positive correlation between BJW and the severity of punishment, consistent with prior research on punitive attitudes toward offenders. Specifically, stronger beliefs in a just world were correlated with higher panel punitiveness, as reviewed in Harvey et al. (2014), indicating that observers who has high BJW may resort to stringent punishment as a strategy for justice restoration (Callan et al., 2017).

Specifically, only observers who has low BJW recommended more severe punishment for high status transgressors, while observers who has high BJW recommended punishment for the same unjust action regardless of the offender’s status. This finding is in line with previous research demonstrating that BJW is a positive psychological resource that could serve to attenuate the negative impact of negative emotions (Bartholomaeus and Strelan, 2019). In line with our Study 2 finding that envy emotions lead to more severe punishment of the high status wrongdoer, BJW may mute the envy emotions thus buffer the hatred to the rich.

In summary, these results provide insights into the influence of BJW on individuals’ punitive attitudes towards wrongdoers of different social status. By demonstrating that higher BJW scores are linked to more equitable punishment recommendations for high-status and low-status wrongdoers, this study adds to the growing literature on the role of BJW in shaping responses to injustice.

## 6 General discussion

### 6.1 Contributions

The present studies contribute to the punishment literature, the stereotype content model (SCM), social status and the belief in a just world (BJW). They extend the literature on punishment by revealing the primacy of emotions in predicting punishment outcomes. By taking group-based envy into account, the present studies highlight two points that have been neglected by previous research on retributive justice. First, the process of punishment judgment is not only related to cognitive inference but also to emotional intuitions, as has been suggested by recent research on moral judgments (Hofmann et al., 2018). Indeed, the process of punishment judgment is not solely directed at the individual who committed the crime but also encompasses their group identity. Thus, blame is both cognitive and social, and is related to the public aspect of punishment, which involves expressing a judgment of blame to another person (Malle et al., 2014). At the same time, this study has expanded the punishment paradigm by adding information about the identity background of the target. During times of growing wealth inequality, feelings of resentment towards the rich may lead people to take social action to express their belief in retributive justice by punishing the rich more severely. This may be based on the expectation that many others will also impose similar punishments, reflecting intersubjective norms.

The present studies also extend the stereotype content model (SCM) by regarding active harm toward high-status groups as more severe punishment. Previous research in the SCM has typically focused on the evaluation of various groups whose information is reduced to their background label (Cuddy et al., 2018a,b,c). Recent research has begun to incorporate additional information into the evaluated group labels, such as misfortune (Dasborough and Harvey, 2017) and counter-stereotype information (Holoien and Fiske, 2013). However, until now, the SCM had not considered the effect of the misbehavior of target groups on the discriminatory behavior they receive. Our research suggests that the misbehavior of target groups may exacerbate discrimination due to the perceived cover of justice motives.

Based on the theoretical and empirical analyzes, our research offers intriguing applications for managing social mentality. Envy is detrimental, while belief in a just world is advantageous. To counteract the negative sentiment towards the wealthy, we should manage envious emotions directed at them and promote just world beliefs within our community. Firstly, the government must make sustained efforts to reduce the wealth gap, thereby alleviating the public’s sense of relative deprivation. Secondly, a positive social mentality should be fostered by intensifying anti-corruption initiatives, which in turn, diminishes envy towards high-status groups. Lastly, individuals should be guided to establish long-term goals, concentrating on socially desirable objectives and methods, ultimately nurturing a belief in a just world.

Building upon previous research, this study extends our understanding on how the belief in a just world (BJW) motivated individuals to maintain justice (Bartholomaeus and Strelan, 2019). When faced with situations that threaten justice, people often take actions to maintain their BJW, such as denigrating innocent victims (Zhou and Guo, 2013). BJW can motivate individuals to act justly, as doing so further reinforces their BJW (Hafer and Sutton, 2016). Individuals with high BJW tend to exhibit equal punishment for the same misconduct by individuals of different status. However, for those with low BJW, their punitive behavior is more influenced by feelings of envy, resulting in more severe punishment for high-status individuals compared to low-status individuals.

### 6.2 Limitation and future directions

While these studies have several strengths, they also have some limitations that future research should address. First, only one item was used to measure intentionality attribution and punishment severity. Second, the same crime behaviors were used to describe scenarios across all three studies. To enhance the generalizability of the current findings, future research should use more systematic and validated scales to measure the attribution of intentionality and the severity of punishment across diverse misbehavior scenarios.

Additionally, further research is needed to compare evaluations of ascribed high status targets with those of achieved high status targets, given their different levels of justice. Although the current research did not find a significant difference in the punishment received by these two groups, more investigation is necessary to fully explore this issue.

In the grand scheme of things, remedying these constraints could yield valuable revelations regarding the impact of intentionality attribution and status on punishment severity, thus deepening our comprehension of the social psychology of punishment.

## 7 Conclusion

The research presented in this article offers valuable insights into the psychological processes underlying the relationship between social status and punishment recommendations. By demonstrating that high-status individuals tend to receive more severe punishments than their low-status counterparts, regardless of whether their status was inherited or self-attained, the findings highlight the influence of status-based biases on people’s judgments and decision-making processes.

In particular, Study 1 replicated the finding that intentionality attributions played a mediating role in participants’ punishment recommendations. This suggests that people may be more inclined to attribute intentional wrongdoing to high-status individuals, possibly because they hold them to higher standards or expect them to be more aware of the consequences of their actions.

Study 2 expanded on this by revealing that envy emotions were a significant factor that partially mediated the relationship between status and punishment recommendations, while intentionality attributions did not play a mediating role in this case. This finding emphasizes the importance of emotional factors in shaping punishment decisions, particularly in situations involving social status. It suggests that people may be more likely to recommend harsher punishments for high-status wrongdoers because they feel envious of their position and success, which could lead to a desire for retribution or leveling the playing field.

Study 3 further elucidated the role of belief in a just world (BJW) in moderating the relationship between status and punishment recommendations. The observation that harsher punishment recommendations for high-status offenders were only found among those who did not endorse BJW implies that individuals who believe the world is fair may be more lenient towards high-status wrongdoers. This offers an intriguing perspective on how people’s beliefs and values can influence their judgments and decisions concerning punishment for individuals of different social statuses.

In summary, this research provides important insights into the roles of social status, emotional factors, intentionality attributions, and belief in a just world in shaping punishment recommendations. These findings contribute to a deeper understanding of the psychological processes involved in evaluating the behavior of individuals with varying social statuses and offer guidance on how to address potential biases and ensure fair treatment for all.

## Data availability statement

The raw data supporting the conclusions of this article will be made available by the authors, without undue reservation.

## Ethics statement

The studies involving humans were approved by Research and Ethics Work Committee of the Department of Psychology, Renmin University of China. The studies were conducted in accordance with the local legislation and institutional requirements. The participants provided their written informed consent to participate in this study. Written informed consent was obtained from the individual(s) for the publication of any potentially identifiable images or data included in this article.

## Author contributions

ZL and FC prepared the publishable manuscript and performed empirical analysis. YW collected the data. QW added valuable theoretical and methodological insights based on his knowledge and expertise regarding the topic. All authors contributed to the article and approved the submitted version.
